# Depth-Dependent Strain Model (1D) for Anisotropic Fibrils in Articular Cartilage

**DOI:** 10.3390/ma17010238

**Published:** 2024-01-01

**Authors:** Syeda Batool, Bradley J. Roth, Yang Xia

**Affiliations:** Department of Physics, Oakland University, Rochester, MI 48309, USA; ssbatool@oakland.edu (S.B.); xia@oakland.edu (Y.X.)

**Keywords:** fixed charged density, anisotropic fibril modulus, fibril pre-strain, 1D axial compression, intra-tissue strains, 1D mathematical model

## Abstract

The mechanical response of articular cartilage (AC) under compression is anisotropic and depth-dependent. AC is osmotically active, and its intrinsic osmotic swelling pressure is balanced by its collagen fibril network. This mechanism requires the collagen fibers to be under a state of tensile pre-strain. A simple mathematical model is used to explain the depth-dependent strain calculations observed in articular cartilage under 1D axial compression (perpendicular to the articular surface). The collagen fibers are under pre-strain, influenced by proteoglycan concentration (fixed charged density, FCD) and collagen stiffness against swelling stress. The stiffness is introduced in our model as an anisotropic modulus that varies with fibril orientation through tissue depth. The collagen fibers are stiffer to stretching parallel to their length than perpendicular to it; when combined with depth-varying FCD, the model successfully predicts how tissue strains decrease with depth during compression. In summary, this model highlights that the mechanical properties of cartilage depend not only on proteoglycan concentration but also on the intrinsic properties of the pre-strained collagen network. These properties are essential for the proper functioning of articular cartilage.

## 1. Introduction

Articular cartilage (AC) is a thin layer of tissue covering the ends of bones in diarthrodial joints. The mechanisms giving cartilage its mechanical resilience involve complex compositional and structural variations across the tissue’s thickness (depth). Several studies [1,2,3,4,5] have demonstrated that such variations are depth-dependent, site-dependent, joint-dependent, age-dependent, and species-dependent. At the compositional level, articular cartilage contains a small number of cells (chondrocytes) surrounded by extracellular matrix (ECM). The major constituents of its ECM are interstitial fluid (water and ions such as Na^+^, Cl^−^, etc.) and macromolecules such as proteoglycans (PGs) and collagens [6]. The most abundant proteoglycan is aggrecan, which is composed of negatively charged side chains of glycosaminoglycans (GAGs). The strongly hydrophilic nature of GAGs can be represented as a fixed charged density (FCD), which is the origin of the osmotic swelling pressure in cartilage [7]. Most of the collagen in articular cartilage is type II, which is fibril collagen. At equilibrium, the osmotic swelling pressure is balanced by the elastic pre-stress in the collagen network and is responsible for tissue stiffness and homeostasis [8,9]. Any imbalance between the PG swelling pressure and collagen fibers’ restraining stress can alter the normal mechanical functioning of the tissue and could be a manifestation or initiation of degradation. 

**Figure 1:**
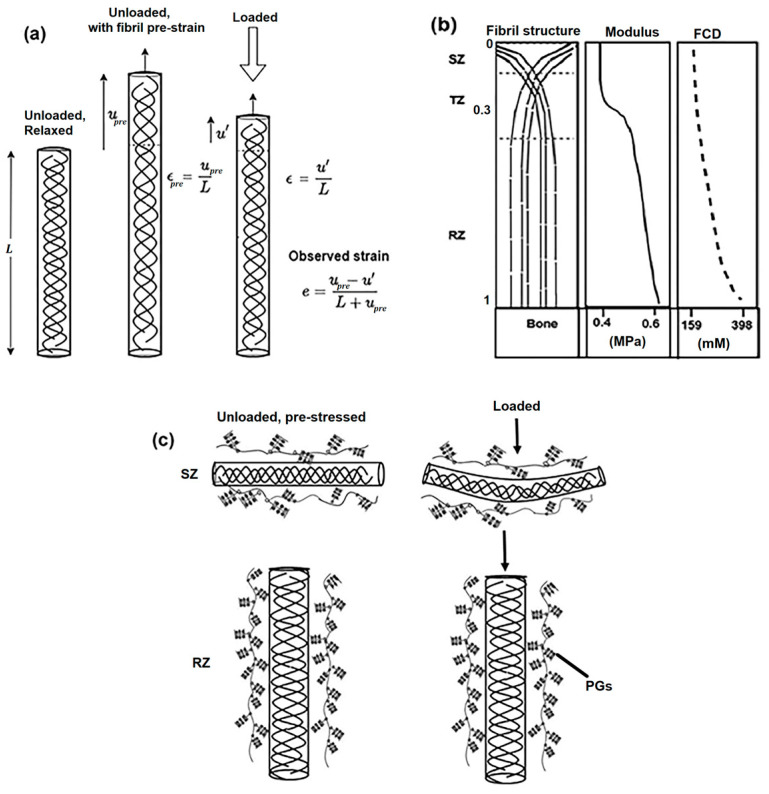
Schematics of the modeling. (**a**) Consider a 1D tissue of length *L* containing collagen fibrils, initially in a relaxed state with zero pre-strain. Introducing a 1D pre-strain $\varepsilon_{pre},$ due to internal FCD, results in a displacement $u_{pre}$. When the pre-stressed tissue undergoes confined compression, the length changes from $u_{pre}$ to $u^{\prime }$, representing the subsequent strain ε. The observed strain (*e*) is the difference between $\varepsilon_{pre}$ and *ε*. (**b**) The depth-dependent variations in articular cartilage with tri-zonal organization of collagen fibers (SZ, TZ, and RZ) (Figure 1b, first), the intrinsic stiffness of the collagen network represented by modulus μ (Figure 1b, middle), and the depth-dependent FCD indicating proteoglycan concentration changes (Figure 1b, third) are shown. These moduli and FCD values represent the selected range of values utilized in our model to find observable strains. (**c**) Collagen fiber schematics in SZ (parallel and thinner, ~50 µm) and RZ (perpendicular and thicker, ~100–200 µm). The PGs entangle around fibers, causing fibril pre-strain dependent on both FCD and μ The fibers in SZ are more compressible than those in RZ under uniaxial loading (third panel). Radial zone collagen fibers undergo no significant structural changes under loading.

PGs in cartilage are considered unstructured; in contrast, collagen in mature healthy cartilage forms a network with a depth-dependent tri-zonal structure (Figure 1b, left). The fibers are parallel to the surface in the superficial zone (SZ) (10–15% of the overall thickness), without any predominant orientation in the transitional zone (TZ) (20–40%), and perpendicular to the surface in the radial zone (RZ) (60–70%). The RZ is often referred to as the deep zone, where radial fibers anchor the cartilage to the subchondral bone [6]. Collagen fibers also differ in size, with fine fibers in the SZ and thicker fibers in the RZ [10,11]. In addition to the depth-dependent structure of the collagen network, the distribution of molecular components (water, proteoglycan, and collagen) is also not homogenous, and varies with depth from the articular surface towards the cartilage–bone interface [6,10,12]. For mature healthy cartilage, the SZ has a lower concentration of PGs and a higher concentration of water compared to the RZ [6]. The depth-dependent variations of the molecular components, in conjunction with the macro and microstructural variations, may control its unique depth-dependent and direction-dependent mechanical deformation response to external loading [13,14,15]. Recently, the role of the collagen fibers’ orientation was predicted to be the primary factor modulating the crack morphology between mature and immature cartilage under indentation loading [16]. The mechanical properties of articular cartilage appear to depend on both the composition and structure of the tissue at an early immature stage [17]. During growth and maturation, structural variations in the collagen network together with compositional changes happen across the tissue depths [2]. The structural changes that occur range from a monolayer to a mixed multizonal layer at the early stages of maturation and finally reach the standard tri-zonal structure found in mature tissues [18]. This tri-zonal structure of collagen fibers and the concentration of macromolecules, especially proteoglycans, are considered to modulate the tissue’s resistance to compressive loading [9,19] through osmotic swelling pressure and electrostatic repulsion between negatively charged PGs.

In the early models of articular cartilage, the tissue was considered to be isotropic, homogenous, and biphasic [20]. Biphasic theory models tissue as a mixture of two phases: a charged porous permeable solid phase (collagen–proteoglycan matrix) and an interstitial fluid phase. Later, a triphasic model [21] of articular cartilage was introduced as an extension of the biphasic model, where negatively charged proteoglycans are modeled to be fixed to the solid matrix, and monovalent ions in the interstitial fluid are modeled as additional fluid phases. According to the triphasic model, the swelling of articular cartilage depends on its fixed charge density distribution, the stiffness of its collagen–proteoglycan matrix, and the ion concentrations in the interstitial fluid. These models, however, lack the structural inhomogeneity and anisotropic properties of extracellular matrix and consider cartilage to be a homogenous, isotropic, and viscoelastic material, which probably does not enable the discrimination of PGs and collagen-dependent mechanical properties leading to a more accurate understanding of cartilage mechanics. To address this issue, several fibril-reinforced models (fibril-reinforced poro-elastic, poro-viscoelastic, and hyperelastic models) have been introduced to include the effect of fibril anisotropy in traditional biphasic and triphasic models [22,23,24,25]. Compared to these models, we use a simple (1D) mixture model, which is complex enough to include the depth-dependent structural inhomogeneity of collagen fibers, including fibril pre-strain and inhomogeneous distribution of FCD; yet, it is simple enough to provide an analytical solution that provides further understanding and insight. Our approach is similar to that proposed by Wang et al. [26], which can predict intra-tissue strains and displacements in conjunction with osmotic swelling pressure and the mechanical properties of the collagen fibril network under one-dimensional (1D) axial compression (confined and under equilibrium) along the depth [8]. 

In this study, the model was computational and solved in MATLAB. The simulation results (intra-tissue strains and displacements) were applied to different scenarios (mature, immature, healthy, and degraded cartilage) and are in accordance with several experimental findings [26,27,28,29,30]. We investigated the role of the collagen network in mature cartilage under 1D axial compression, where the fibril network was in pre-strain conditions (due to osmotic swelling) that depended on both the concentration of PGs (FCD) and the intrinsic stiffness of the collagen fibers’ network. We modeled the collagen as a series of linear springs with intrinsic stiffness denoted by an anisotropic mechanical modulus (μ) that depends upon the depth-dependent orientation of the tri-zonal architecture of collagen fibers in articular cartilage. We assumed that the collagen network was stiffer when stretching parallel to the length of its fibers (in the RZ) than perpendicular to it (in the SZ) (Figure 1b, middle). We will show that the intrinsic structural inhomogeneity of collagen fibers and the inhomogeneous distribution of FCD caused an anisotropic pre-strain in the tissue and increased the tissue’s resistance against axial loading, specifically in the radial zone. In particular, we intend to examine the relative importance of collagen networks’ intrinsic stiffness towards osmotic swelling, contributing to the response of cartilage to compression.

## 2. Materials and Methods

The fundamental assumptions used in this study are described in Figure 1a,b. Cartilage is assumed to be compressible and permeable (water flows in and out). Initially, before any osmotic swelling takes place, the unstretched and unloaded tissue (stress τ=0) has a cross-sectional area A, a length L, and an FCD concentration $C_{c}$ (Figure 1a, left). We model the collagen network as a series of linear springs with intrinsic stiffness denoted by a mechanical modulus (μ). Due to the osmotic pressure caused by the FCD, the tissue swells, causing a pre-strain $\varepsilon_{pre}$. Let $u_{pre}$ be the distance it displaces in the radial direction, so strain $=\varepsilon_{pre}=\frac{u_{pre}}{L}$ (Figure 1a, middle). This swelling causes the effective concentration of FCD to drop from its unstrained value $C_{c}$ (assuming that the number of molecules remains the same but the volume changes) as the tissue is diluted by additional water, giving a strain-dependent concentration,

(1) $$C=\frac{C_{c}}{\left(1+\varepsilon_{pre}\right)}\;.$$

When the tissue is loaded under uniaxial compression (τ≠0), let $u^{\prime }$ be the distance it displaces from the initial unstretched, unloaded state. The resultant strain will be $\varepsilon =\frac{u^{\prime }}{L}$ (Figure 1a, right). It should be noted that *L* is the initial reference length with zero pre-strain (a nonphysiological condition). The observed strain, e, is the net difference between displacements ($u_{pre}$ and $u^{\prime }$) over the pre-compression length (L + $u_{pre}$). 

To model intra-tissue strains and displacements at equilibrium, when the osmotic swelling stress in cartilage is balanced by the elastic stress exerted by a pre-stressed collagen network [8], the total stress (τ) in the tissue can be represented as the osmotic stress ($\tau_{FCD})$ minus the elastic stress of the collagen network (*τ_CN_*) [8,31],

(2) $$\tau =\tau_{FCD}-\tau_{CN},$$

(3) $$\tau =CkT-\mu \varepsilon_{pre}.$$

For simplicity, a 1D analysis of cartilage occurs in confined compression, which means that the tissue is not allowed to undergo displacements or strains in the direction perpendicular to its depth, as if the tissue was confined between rigid walls. Furthermore, we assume that all tissue properties depend only on depth and do not depend on their position perpendicular to the depth. It follows that the normal stress τ (perpendicular to the articular surface) is independent of the depth (the tissue is in static equilibrium) even if the strain and mechanical properties vary with depth. These assumptions are reasonable because cartilage’s thickness is much less than the width of a typical joint. 

The total normal stress can be modeled as Equation (3), where $\tau_{CN\;}=\mu \varepsilon_{pre}$ is the elastic stress exerted by the collagen network, and $\tau_{FCD}=CkT$ is an osmotic term that depends on the concentration of FCD C, the Boltzmann constant k, and the absolute temperature T. In this expression, C must be given in molecules per unit volume; if given in moles per unit volume, the Boltzmann constant k should be replaced by the gas constant *R*. For simplicity, we assume that the elastic stress–strain relationship is linear. 

When pre-strained tissue is under zero external stress (τ=0), Equation (3) can be written as

(4) $$\frac{C_{c}kT}{\mu }=\varepsilon_{pre}\left(1+\varepsilon_{pre}\right).$$

Solving Equation (4) quadratically gives the pre-strain as

(5) $$\varepsilon_{pre}=\frac{-1+\sqrt{1+\frac{4C_{c}kT}{\mu }}}{2}\;.$$

When tissue is stressed under uniaxial compression (τ≠0), the observed strain, e, is

(6) $$e=\frac{u_{pre}-u^{\prime }}{\left(L+u_{pre}\right)}\;$$

or

(7) $$e=\frac{\varepsilon_{pre}-\varepsilon }{\left(1+\varepsilon_{pre}\right)}.$$

From Equation (3), the total stress can be written as

(8) $$\tau =\frac{C_{c}}{\left(1+\varepsilon \right)}\;kT-\mu \varepsilon .$$

Rearranging Equation (8) and using Equation (4) gives

(9) $$\frac{\tau }{\mu }=\frac{\varepsilon_{pre}\left(1+\varepsilon_{pre}\right)}{\left(1+\varepsilon \right)}-\varepsilon .$$

Solving Equation (9) for ε using the quadratic equation gives

(10) $$\varepsilon =\frac{-\left(1+\frac{\tau }{\mu }\right)+\sqrt{{\left(1-\frac{\tau }{\mu }\right)}^{2}+4\left(\varepsilon_{pre}{}^{2}+\varepsilon_{pre}\right)}}{2}\;.\;$$

The observed strain can be calculated using Equations (5), (7), and (10), as follows:

(11) $$e=\left(\frac{\varepsilon_{pre}}{1+\varepsilon_{pre}}\right)+\left\{\frac{\left(1+\frac{\tau }{\mu }\right)-\left(\sqrt{{\left(1-\frac{\tau }{\mu }\right)}^{2}+4\left(\varepsilon_{pre}{}^{2}+\varepsilon_{pre}\right)}\right)}{2\left(1+\varepsilon_{pre}\right)}\right\}.\;$$

In the above equations, if $\frac{\tau }{\mu }$ is equal to $\varepsilon_{pre}\left(1+\varepsilon_{pre}\right),$ then ε=0, implying that the cartilage is unstretched. Because the cartilage network cannot resist compression, our model no longer holds if $\frac{\tau }{\mu }>\varepsilon_{pre}\left(1+\varepsilon_{pre}\right)$.

To gain further insight, consider the approximation τ<<μ in Equation (11). In that case, the stress–strain relationship reduces to

(12) $$\tau =\mu \left(1+2\varepsilon_{pre}\right)e;\;$$

so, the effective modulus $\mu_{eff}$ is

(13) $$\mu_{eff}=\mu \left(1+2\varepsilon_{pre}\right).\;$$

During compression and under small stresses, the effective modulus of the tissue is governed by the contributions from the intrinsic stiffness μ of the collagen network and the ratio $\frac{C_{c}kT}{\mu }$. It should be noted that, by assuming τ<<μ, we are merely examining a limiting case to gain insight into the behavior of tissue at smaller stresses. For larger and more realistic stresses this approximation will not hold, and the more complicated Equation (11) must be used to determine the stress–strain relationship. 

The fibril pre-strain $\varepsilon_{pre}$—which depends on the FCD ($C_{c})$ and the intrinsic stiffness of the collagen network (μ), both of which vary with depth (Figure 1b, middle and right)—can also be non-uniform and depth-dependent and depends on variations of these two parameters (Equation (5)). The observed strain (*e*) under an applied load can be calculated using Equation (11) considering uniaxial compression (confined, constant load, and equilibrium) and will be non-uniform along the depth of the tissue.

### 2.1. Depth-Wise Variation in Fiber Orientation

Non-calcified, healthy, mature cartilage is commonly sub-divided into three structural zones (SZ, TZ, and RZ), where the lower/deeper end of the radial zone is anchored to the subchondral bone. The collagen fibers in different zones have preferred orientations, as shown in Figure 1b (left). The tissue is divided into three zones based on the depth-dependent angle profile θ of the collagen fibers [32]

(14) $$\theta \;\left(y\right)=\frac{\pi }{4}\left[\tan h\left(\frac{y-m1}{m2}\right)+1\right]\;.$$

To represent a typical tri-zonal architecture of mature cartilage with depth *L* = 1 mm, we take m1 = 0.3 mm and m2 = 0.09 mm, where θ is the angle between the fiber direction and the plane of the articular surface (θ ~ 0 in SZ and $\theta \;\sim \;\frac{\pi }{2}$ in RZ); *y* refers to the depth of the tissue with zero at the articular surface; m1 refers the center of TZ or the location of most random fibers, and m2 is the length scale over which the fibers are changing their orientation. The thickness of each zone can be determined using Equation (14), employed by Xia et al. [32]. 

### 2.2. Depth-Wise Variation in FCD and µ

The depth-wise increase in FCD (Figure 1b) is incorporated into the model using

(15) $$C_{c}\left(y\right)=C_{0}\exp\left(\frac{y}{r}\right)\;,\;$$

where $C_{0}$ is the concentration of FCD near-surface (y = 0), and r is the rate at which the concentration varies with the depth. The typical value of FCD ranges from 50 to 250 mM for human cartilage [33,34]. The calculation of the FCD used in the simulations is based on a depth-dependent GAG concentration [35,36] for mature canine cartilage, assuming two moles of negative charge per mole of chondroitin sulfate (one sulfate and one carboxylate), which has a molecular weight of 502.5 g/mole. These values (159–398 mM) lie within the range reported for articular cartilage FCD in the literature [5,14,36,37,38], as shown in Figure 1b (right). The concentration of FCD, which depends on PG concentration, is not only depth-dependent but also varies among species, type, and locations in the joint, and it has also been reported to change during growth and maturation [17]. Therefore, the choice of $C_{0}$ and r would be sample-specific to fit the simulation results.

Another assumption in our model was that the intrinsic stiffness of the collagen network (denoted by modulus μ) in articular cartilage is anisotropic and depth-dependent (it varies with fiber orientation along its depth), as shown in Figure 1b (middle). We assumed that the collagen fibers are stiffer parallel to their length (in the RZ) than perpendicular to it (in the SZ). The stress–strain relationship in anisotropic tissue has been derived previously [39]. For the 1D case, the collagen network modulus can be written as

(16) $$\mu \left(y\right)=\mu_{0}+\mu_{1}\sin^{4}\theta \;,\;$$

where $\mu_{0}$ is the modulus of the collagen network near the surface in the SZ (θ=0), and $\mu_{1}$ is the additional contribution to the modulus in the RZ (θ=π/2). (A derivation of Equation (16) is given in Appendix A). Considering 0.4 MPa for SZ and 0.6 MPa for RZ, the corresponding $\mu_{0}$ and $\mu_{1}$ are given as

(17) $$\mu_{0}=0.4\times 10^{6}\;\left(\mathrm{Pa}\right)$$

(18) $$\mu_{1}=0.08\times 10^{6}\;\exp\left(\frac{y}{0.00099}\right)\;,$$

where the factor 0.00099 was obtained by assuming 1 mm length of tissue as the distance between the SZ and the RZ and using the 0.4 to 0.6 MPa difference of collagen stiffness between the two regions. The values we have used (0.4 MPa for SZ, 0.6 MPa for RZ) are comparable to the values of solid matrix stiffness of immature bovine cartilage from carpometacarpal joints [26].

In general, when an external force deforms an elastic body, the resistance to deformation is called stiffness. It could be a function of material properties, material orientation, geometric dimensions, loading directions, type of constraint, and choice of spatial region, where loads and constraints would be applied. The fibril pre-strain $\varepsilon_{pre}$ in our model is influenced by both FCD ($C_{c}$) and the intrinsic stiffness of collagen network μ, as described in Equation (5). 

To determine the order of magnitude for the value of the pre-strain, let $C_{c}$ = 200 mM and μ = 0.5 MPa (replace k with *R* in Equation (5) because we are using $C_{c}$ in mM for this calculation). The calculated pre-strain value using Equation (5) is 0.63. Therefore, there is significant stretching of the collagen network in the pre-strain condition. We further consider that both FCD and intrinsic stiffness vary with depth, as indicated in Equations (15) and (16), resulting in a non-uniform and depth-dependent pre-strain. We are interested in exploring the role of depth-varying fibril pre-strains along with FCD in resisting the axial compression of articular cartilage.

## 3. Results

### 3.1. Nonlinear Strain (e vs. τ Relationship) 

Equation (11) implies that the observed stress–strain relationship is nonlinear when the cartilage is compressed, even if the collagen stress–strain relationship is linear (defined in the text by Equation (2)). To appreciate the relative influence of collagen and proteoglycan, assume $\varepsilon_{pre}<<1$ and τ<<μ. The effective modulus can be obtained by simplifying Equations (11) and (12) as

(19) $$\mu_{eff}=\mu +2C_{c}kT\;,\;$$

which is the sum of a term relating to the intrinsic stiffness of the collagen network and a term relating to the contribution of FCD (twice the osmotic term). As the stress becomes larger, the observed strain *e* in Equation (11) becomes independent of stress. If τ becomes larger than $\mu \varepsilon_{pre}\left(1+\varepsilon_{pre}\right),$ the collagen network alone cannot support compressive stress, and our model no longer holds (loss of pre-strain corresponds to ε=0).

**Figure 2:**
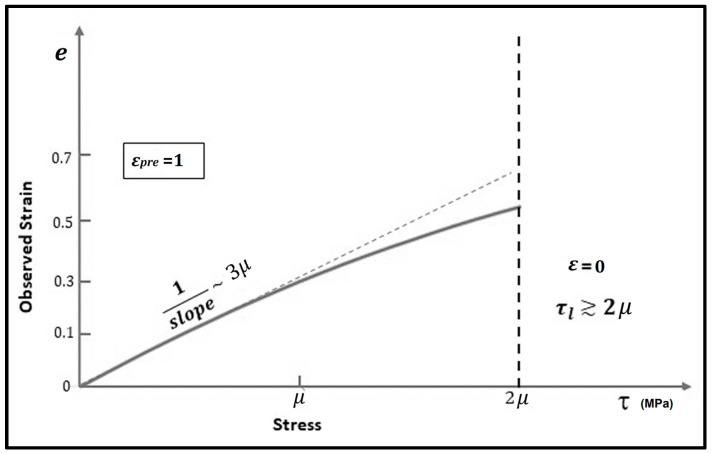
The relationship between applied stress τ and observed strain *e* for an assumed pre-strain $\varepsilon_{pre}$ = 1, using Equation (11).

Figure 2 shows the *e* versus *τ* relation calculated using Equation (11) for *ε_pre_* = 1. For small, applied compressive stresses (τ), the stress vs. strain relation is linear, but, with increased applied stress, it tends to be nonlinear (*τ* becomes larger than *μ*). If τ > 2*μ*, the collagen loses its pre-stretch (*ε* = 0).

### 3.2. Effect of Variations of FCD and µ on Strain

**Figure 3:**
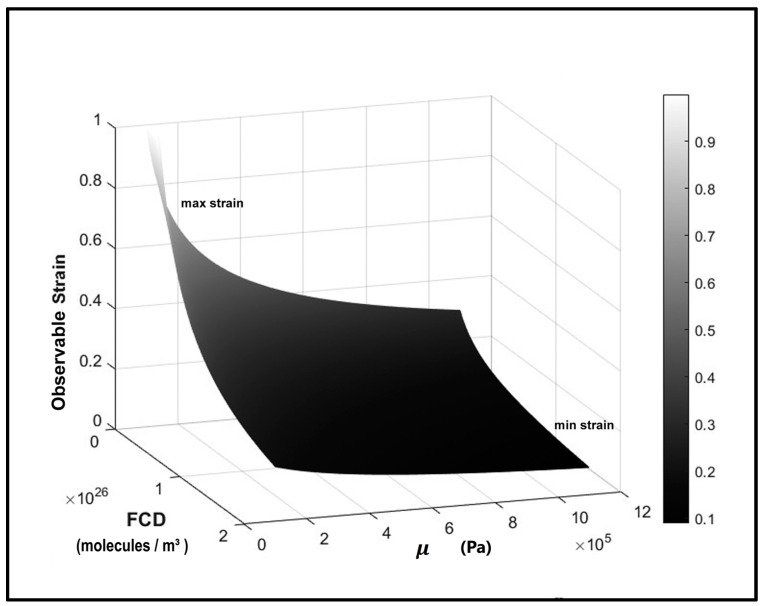
Effect of varying FCD and μ on simulated strains for a given stress (τ = 0.2 MPa). The values of FCD given in mM were converted to molecules/m^3^.

The observed strains (Equation (11)) were simulated in Figure 3 for the given ranges of FCD and μ as two independent parameters. When both FCD and μ are small, the predicted strains are large (an observed trend for the SZ of compressed cartilage), while, for larger FCD and μ, the strains appear smaller (RZ trend).

### 3.3. Depth-Dependent Strain

**Figure 4:**
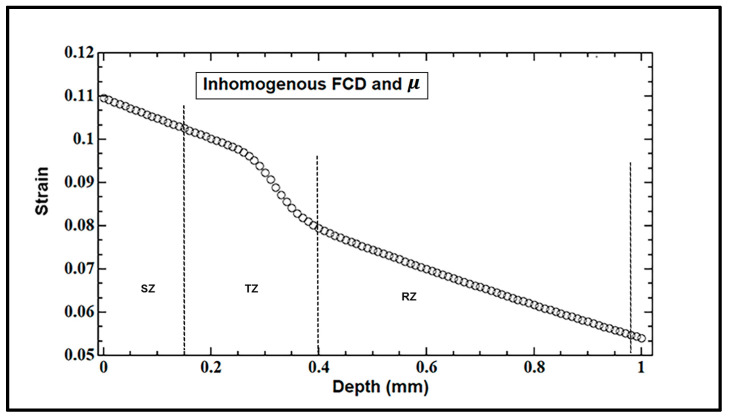
The simulated observed strain *e* over depth when both FCD and μ are varying with depth. The tri-zonal tissue has a length L = 1 mm (m1 = 0.3 mm, m2 = 0.09 mm) and, under the applied stress, τ= 0.1 MPa.

The simulated tissue strain is obtained by introducing depth-dependent parameters FCD and *μ* (Equations (15) and (16)) considering mature healthy cartilage with *L* = 1 mm, m1 = 0.3 mm, and m2 = 0.09 mm (Figure 4). The FCD near the surface before a stress was applied $(C_{0}=159\;\mathrm{mM}$) was obtained from experimental studies [11,12] and varied with depth (r = 0.9 mm in Equation (15)). The values of the FCD used in these simulations are based upon the depth-varying GAG concentration (from 159 mM at the surface to 398 mM at radial zone) reported for mature canine humeral cartilage obtained from different experimental studies [11,12,33] (MRI, CT, and biochemical); all of these studies provided similar values and are strongly correlated. For a preliminary analysis, the intrinsic modulus of the collagen fibers near-surface was 0.4 MPa, which increases to 0.6 MPa in deep regions, as described above in Equations (16)–(18).

The simulated intra-tissue strain decreases non-homogenously through the depth (τ = 0.1 MPa), as shown in Figure 4. A positive stress or observed strain corresponds to a compression in our model. The tissue strain is highest near the articular surface and decreases (~50%) from the SZ to the deep RZ and in the middle ‘kink’ occurring in the TZ at a depth between 0.2 and 0.4 mm caused by the rotation in the fibers’ direction.

### 3.4. Force vs. Intra-Tissue Displacement Relation

**Figure 5:**
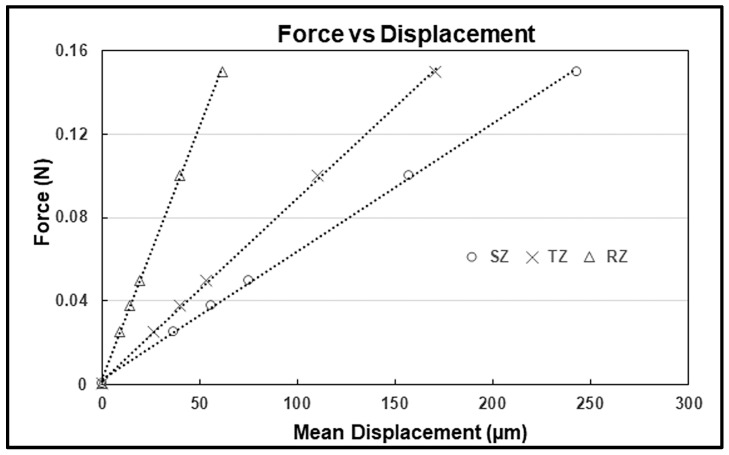
The equilibrium force vs. intra-tissue displacement plots for three pre-defined zones (SZ, TZ, and RZ).

The strains averaged over the thickness of each zone (as determined by Equation (13)) are represented as zonal strains. The intra-tissue displacement for each zone of tissue can be obtained by integrating the depth-varying observed strain *e* along the depth and then averaging the displacement results over the thickness of each zone. In general, the RZ has the least mean intra-tissue strain and displacement compared to the SZ and TZ, consistent with what is observed experimentally [14,27]. Figure 5 represents the relationship between applied force (stress) and simulated intra-tissue displacements for three zones (SZ, TZ, and RZ) of a thin rectangular sample tissue, compressed (surface-to-surface) by increasing stress (τ) in discrete steps (stress relaxation).

The applied stress (τ) can be converted into force (F) as F=τA, where A is the surface area (A = thickness × width) in contact with the loading platen (0.25 mm^2^). The thickness of the tissue is assumed to be ~120 µm; the width is ~2 mm, and it is compressed with a thin glass platen (thickness ~100 µm). These simulated zonal relationships between force and displacement agree with the known mechanical response of cartilage under compression and are comparable to the experimental measurements of the intra-tissue displacements in mature canine humeral articular cartilage [27] (linear model, 10–20% error). The total depth of the tissue used in the simulation is 0.55 mm, compared to the cartilage depth of 0.50 mm mentioned in the experimental study above [27]. The rest of the parameters are the same as described above to simulate a depth-dependent strain for mature tri-zonal cartilage.

### 3.5. Effective Modulus for Cartilage Zones (Stress vs. Strain)

**Figure 6:**
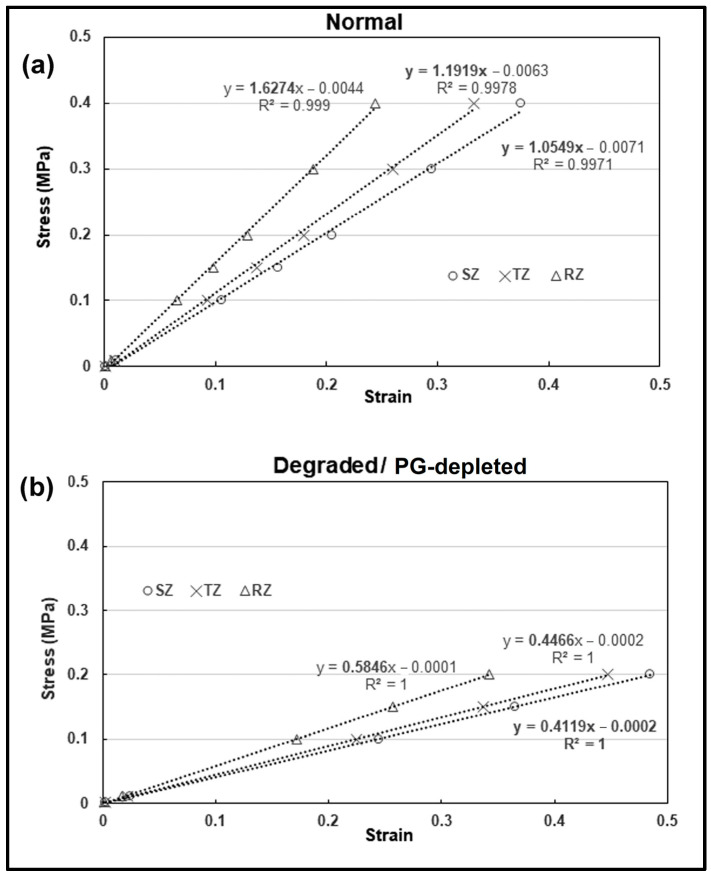
The effective compressive modulus, $\mu_{eff}$, in three zones of cartilage. (**a**) The effective compressive modulus, $\mu_{eff}$, in three zones in a simulated ‘mature and healthy’ cartilage, using the stress-strain relationship (slope of linear fit represents the $\mu_{eff}$). For the SZ the modulus is ~1 MPa and it increases to ~1.6 MPa in the deep region (RZ). (**b**) The effective compressive modulus, $\mu_{eff}$, in three zones in a simulated ‘degraded’ cartilage that has a reduced PG content. Loss of PG or FCD results in an increase in the intra-tissue strains (~50%) for the given stress, and consequently a decrease in effective modulus (slope of linear fit) for each zone.

Figure 6 presents the stress vs. strain relation for three zones of normal (Figure 6a) and PG-depleted or -degraded tissue (Figure 6b). For the normal tissue (Figure 6a), the parameters (FCD, *μ*, m1, m2) are the same as described earlier (Figure 4 and Figure 5). The mean strain appears to increase linearly with increasing stress for all three zones. The slope of the linear fit represents the effective modulus $\left(\mu_{eff}\right)$ of each zone. The effective modulus increases with depth, from ~1 MPa in SZ to 1.6 MPa (smaller than what is reported by other studies) in the deep region (RZ). The discrepancy in the RZ modulus was attributed to the choice of elastic stiffness (μ) for the RZ fibers (which could be higher than 0.6 MPa). With the adjustment of μ (at the deep region, RZ) in the range between 1 and 1.5 MPa instead of 0.6 MPa, the simulation produced similar depth-dependent intra-tissue displacements and intra-tissue strains when compared with the data reported for the human femoral head cartilage [14] (τ = 0.01–0.4 MPa). The value of μ was chosen by trial-and-error. The FCD used in the simulation is the same as the one mentioned before $\left(C_{0}=159\;\mathrm{mM},\;r=0.9\;\mathrm{mm}\right)$.

The loss of PG can cause a loss in FCD, which can be attained through different enzymatic digestions (e.g., trypsin) and is the characteristic feature of degraded tissue. As a result, tissues can experience an abnormal increase in intra-tissue strains and displacements for a given otherwise-normal stress. Figure 6b presents the stress vs. zonal strain relation for the three zones of the PG-depleted or -degraded tissue, with μ the same as normal (0.4–0.6 MPa), assuming no change in the intrinsic properties of the collagen fibers. The loss of PG or FCD results in a decrease in pre-strain $\varepsilon_{pre}$, an increase in the intra-tissue strains (~50%) for the given stress, and, consequently, a decrease in the effective modulus (slope of linear fit) for each zone (60% for the SZ, 62% for the TZ, and 64% for the RZ). The depth-wise loss of FCD was introduced in the model by adjusting the SZ’s FCD ($C_{0}$) from 159 mM to 1.59 mM (~99% decrease) and, in the RZ, from 398 mM to 79.6 mM (~80% decrease), while the μ is the same as that used for the normal case. The effective modulus decreases ~50% for each zone compared to normal native tissue (Figure 6a) and is consistent with experimental findings that the selective enzymatic digestion of PGs can cause a significant drop in the elastic compressive modulus of articular cartilage [40,41].

### 3.6. Strains between Homogeneous and Inhomogeneous Tissues

**Figure 7:**
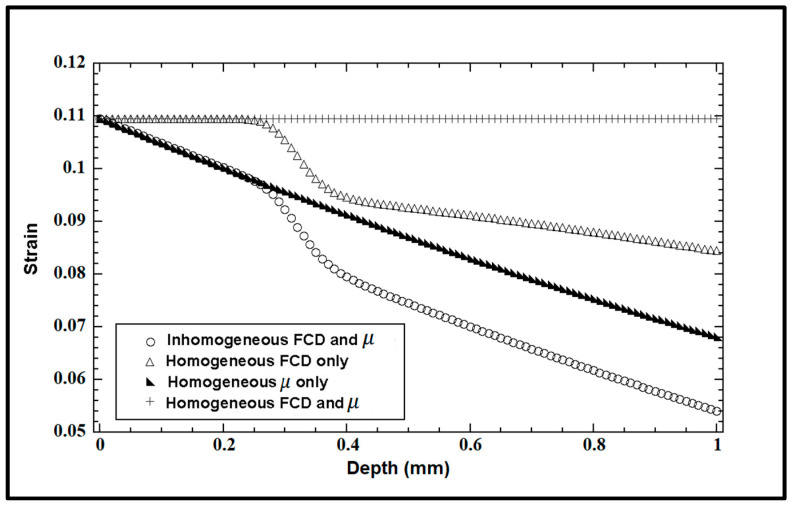
The simulated strains for the given stress (τ= 0.1 MPa) at different parameter conditions (FCD, μ, homogeneous and non-homogeneous) as a function of tissue depth.

Figure 7 presents several intra-tissue strains as a function of the tissue depth, simulated for a given stress (τ= 0.1 MPa) under different parameter conditions (FCD, μ) between homogenous (e.g., neonatal cartilage) and inhomogeneous (e.g., healthy, mature) cartilage, with *L* = 1 mm. For comparison, a constant FCD ($C\;\sim \;C_{0}=159\;\mathrm{mM}$, r → ∞) and a constant intrinsic modulus of collagen network ($\mu \;\sim \;\mu_{0}=0.4\;\mathrm{MPa},\;m1=0,\;m2\to \infty$) were used for the homogenous case (no depth variations), keeping the rest of the parameters the same as before.

The homogeneous distributions of both FCD and μ along the depth generated constant strain values. The condition of a constant intrinsic modulus of the collagen network can be attained in neonatal cartilage [17] or nasal cartilage [42,43] with a uniform fibril organization where most fibers align parallel to an external surface, i.e., θ = 0. The inhomogeneous case of both FCD and μ used the same parameters as those used to simulate the depth-dependent strain in Figure 4. By comparison, the intra-tissue strain in articular cartilage decreases significantly with depth in the middle and deep zones (0.4–1 mm) of the tissue when it has non-homogenous distributions of both FCD and μ (inhomogeneous FCD and μ), which are characteristic of mature cartilage tissue.

## 4. Discussion

Articular cartilage has unique depth-dependent structural and material anisotropy. A better understanding of anisotropy in biological tissues is important for predicting material properties and designing novel engineering structures. Considering this tissue’s structural anisotropy, the effect of a force’s loading direction can be explored through the examination of its structural and material characteristics in the loading direction, related mostly to the anisotropic structure of collagen networks in the cartilage. 

The role of collagen fibers in articular cartilage under compression has been studied extensively [44,45,46,47], and depth-dependent deformation and fibril reorientation responses have been reported [14,46]. In mature healthy cartilage, the RZ exhibits a stronger resistance to compression than the SZ and the TZ. Various theoretical models [48,49,50,51] (e.g., isotropic elastic, isotropic biphasic or triphasic, fibril-reinforced poro-elastic, etc.) have been developed to explain the depth-dependent compressive response of articular cartilage.

In this study, we have presented the simulation results for 1D loading (normal to the articular surface) only. The model uses the structural anisotropy of the fibril network to explain the depth-dependent mechanical compressive properties of the tissue. We intended to explore the role of the zonal structure of pre-stressed collagen fibers in the loading response of cartilage and provide some insights to design advanced anisotropic fibril-reinforced models of cartilage. Our model emphasized the structural arrangement of the collagen network that influences the compressive nature of cartilage and we examined its nonlinear strain response. A critical assumption in our model is that the collagen network is stiffer along its longitudinal axis (RZ) than its perpendicular axis (SZ), as illustrated in Figure 1c. This assumption is introduced into our modeling as a fibril modulus that varies with fiber orientation along the depth of the tissue (Figure 1b middle). When combined with a depth-wise increase in proteoglycan content (represented by the FCD; Figure 1b right), the collagen networks in the RZ appear to be more resistant (minimal observed strain) to uniaxial compression than those in the SZ and TZ (maximum observed strain). Although the exact role of fibrillar mechanics in the collagen network is not clear, an increase in fibrillar pre-strain may be associated with an increasing fibrillar D-period (nanoscale structure) with depth [52], which, in turn, may be controlled by the known depth-wise variation in proteoglycan content and the subsequent swelling pressure.

The fibril pre-strain in our model depends on the intrinsic stiffness μ of the collagen network (anisotropic; higher longitudinal than transverse) and FCD. Since FCD varies with cartilage depth in addition to fibril orientation, our simulated fibril pre-strain was higher in the RZ (FCD and μ higher) than in the SZ (FCD and μ lower). The effective modulus for the SZ qualitatively agrees with experimental results [27], while the RZ modulus (1.6 MPa) is significantly smaller than what the experimental study referenced found (~6 MPa). We hypothesize that this difference arises because of the choice of elastic stiffness (μ) for the RZ fibers (it could be higher than 0.6 MPa) and not the FCD, since the FCD can be measured more easily and accurately. This hypothesis is supported by Chen et al. [14], who highlight the role of other factors (unknown) besides FCD at different depths of tissue to explain the marked increase in the confined compression modulus of human femoral head articular cartilage in the deep radial zone (RZ). 

When this pre-stressed structure was compressed perpendicularly to the articular surface, the simulated intra-tissue displacement and observed strain decreased with depth below the articular surface (minimal observed strains appear for the RZ). The simulation results are in agreement with several macro-scale experimental findings of a higher compressive modulus in the RZ than the SZ [26,27,28,29] in articular cartilage, except for some which reported the opposite trend [53]. We speculate that the model prediction of a higher compressive modulus in the RZ (smaller strains, strain hardening) can be explained if we assume that the intrinsic stiffness of the collagen network reaches a maximum when the tissue is mature (closure of the growth plate). If the tissue is immature (growth plate open), we hypothesize that the intrinsic stiffness of the collagen network in the RZ could be smaller than in the mature tissue, even if both have radial fibers in the RZ. A recent study has quantified the structural differences between the immature and mature articular cartilage of rabbits using microscopic MRI and polarized light microscopy [54], and we speculate that, in the case of cartilage, the intrinsic stiffness of the collagen network arises not only because of their orientation but also because of the presence of prototypic fibrils that can fuse or interlace into thicker fibrils, cross-link, etc., particularly in the RZ [11,53,55], which increase as the tissue matures. This inter-fibril organization stabilizes the collagen assembly by the intramolecular and intermolecular linkages. This hypothesis can be tested by means of comparison of the compressive properties of cartilage at different stages of growth and maturation, which involves structural adaptations including collagen fiber architecture.

A polarized Raman spectroscopy study combined with the nanoindentation technique [56] reported a marked increase in the elastic modulus in the deeper part of the cartilage tissue and no correlation between its composition and the local mechanical modulus, which emphasized the contribution of ECM microstructural anisotropy to the tissue’s compressive ability. We suggest that the local mechanical modulus of the anisotropic articular cartilage should not only depend upon the concentration of macromolecules (PG, collagen) but also the intrinsic structural properties of the extracellular matrix (ECM microstructure), including cross-linking, a shift in fiber orientation, mutual organization, etc., of the collagen fibers, which all influence fibrils’ pre-strain and, consequently, the compressive stiffness.

In native mature cartilage, fibrils are pre-strained by the proteoglycans almost to their maximum limit, while this pre-strain is lost upon enzymatic digestion (e.g., trypsin, chondroitinase ABC) [52]. This loss can lead to a significant decrease in tissue compressive modulus [57], but it has also been reported that PG depletion should not affect some intrinsic material properties of collagen fibers (e.g., cohesive strength, orientation, fiber-to-fiber interaction, length, width, etc.), which depend upon the structural linkages within the fibril meshwork itself [57] and could be a maturation-related phenomenon [58]. So, when simulating the observed strains in PG-depleted cartilage (Figure 6b), we assumed that the loss of PG should not change the intrinsic stiffness of the collagen network. The loss in fibril pre-strain in our case is due to the loss of FCD (PG) not the μ.

Our model can be used to estimate the mechanical response of both mature and immature articular cartilage if the selection of parameters is made accordingly. The actual developmental changes of articular cartilage in animal growth involve complex mechanical and biochemical changes dependent upon compositional and structural changes of the extracellular matrix macromolecules, including PGs and collagen fibers. We speculate that the intra-tissue strains and displacements during uniaxial compressive loading could be higher in the deep zone of immature or neonatal tissue, which is devoid of radial fibers and possesses approximately a non-structural organization with most fibers parallel to the articular surface. So, structurally, a homogeneous tissue (neonatal) with no change in the intrinsic stiffness of the collagen network (0.4 MPa) through depth and with no change or increase in the FCD with respect to the depth (Figure 7) will have a smaller compressive modulus than a structurally inhomogeneous tissue (mature), which is consistent with Gannon et al.’s [59] findings. In general, it is useful to study the local strains in the tissue at different stages of maturation as they may regulate cell function and metabolism [60].

## 5. Assumptions and Limitations

The applied stress in our model is global stress, and the slope of a linear fit to stress vs. strain does not represent a real quantification of zonal modulus. In addition, the relationship between the stress and the mean zonal strain appears linear within the model limits (smaller strains) and our choice of parameters. If a nonlinear relationship exists between stress and strain in the tissue, the predicted zonal modulus will change.

The compositional and ultrastructural features of cartilage lead to inhomogeneity and anisotropy in mechanical properties; sometimes, it is difficult to distinguish the independent effect of one rather than the other. The functional properties of cartilage depend on its non-homogeneous extracellular matrix’s molecular composition and structure. Besides normal depth-wise variations in composition and structure, the proteoglycan and collagen vary significantly in content during growth and maturation, along with strong structural adaptations. Currently, the clear answer to the question of what component of collagen fibers determines the intrinsic stiffness (resistance to pre-stretch in case of osmotic swelling, i.e., in a pre-strain condition) and how it varies along with the depth is not clear. In general, the intrinsic properties of the collagen network can depend on its collagen content, the type, size/diameters of the collagen fibers, crosslinking, fiber-to-fiber interaction, fiber orientation, and strength of the macromolecular interactions modulated by the collagen network. In this study, we focus on the behavior of the entire collagen network. The stiffness and strength of the network may be different from the stiffness of individual collagen fibers [61,62]. It should ideally be independent of GAG/FCD concentration.

It has also been reported that articular cartilage obtained from different joints of the same animal can be different in its composition, and, similarly, the cartilage obtained from weight-bearing and non-weight-bearing regions of the same joint can offer different stiffness under the same loading conditions. Therefore, for the theoretical analysis and comparisons of compressive behavior under the same loading conditions, the choice of parameters, including collagen network stiffness, depends upon the sample and its conditions under consideration, thus requiring sophisticated and careful measurements of biochemical and biomechanical gradients.

## Data Availability

Data are contained within the article.

## Appendix A

Derivation of Equation (16)

Assume that the stress and strain consist of a part that is isotropic and a part that is anisotropic. For the anisotropic part, the stress in the fiber’s frame of reference (with the *y*′ axis along the fibers) is related to the stress in the tissue’s frame of reference (with the *y* axis varying along the depth in the cartilage) by

$$\tau_{xx}=\tau_{xx}^{\prime }\cos^{2}\phi +\tau_{yy}^{\prime }\sin^{2}\phi -\tau_{xy}^{\prime }2\sin\phi \cos\phi$$

$$\tau_{yy}=\tau_{xx}^{\prime }\sin^{2}\phi +\tau_{yy}^{\prime }\cos^{2}\phi +\tau_{xy}^{\prime }2\sin\phi \cos\phi$$

$$\tau_{xy}=\left(\tau_{xx}^{\prime }-\tau_{yy}^{\prime }\right)\cos\phi \sin\phi +\tau_{xy}^{\prime }\left(\cos^{2}\phi -\sin^{2}\phi \right),$$

where *ϕ* is the angle between the fibers and the *y*-axis. In the fiber’s frame of reference, Hooke’s law is

$$\tau_{yy}^{\prime }=\mu \varepsilon_{yy}^{\prime },$$

with $\tau_{xx}^{\prime }=\tau_{xy}^{\prime }=0$. So,

$$\tau_{xx}=\mu \;\varepsilon_{yy}^{\prime }\sin^{2}\phi$$

$$\tau_{yy}=\mu \;\varepsilon_{yy}^{\prime }\cos^{2}\phi$$

$$\tau_{xy}=-\mu \;\varepsilon_{yy}^{\prime }\cos\phi \sin\phi .$$

The strain in the fiber’s frame is related to the strain in the tissue frame by

$$\varepsilon_{xx}^{\prime }=\varepsilon_{xx}\cos^{2}\phi +\varepsilon_{yy}\sin^{2}\phi +\varepsilon_{xy}2\sin\phi \cos\phi$$

$$\varepsilon_{yy}^{\prime }=\varepsilon_{xx}\sin^{2}\phi +\varepsilon_{yy}\cos^{2}\phi -\varepsilon_{xy}2\sin\phi \cos\phi$$

$$\varepsilon_{xy}^{\prime }=\left(\varepsilon_{xx}-\varepsilon_{yy}\right)\cos\phi \sin\phi +\varepsilon_{xy}(\cos^{2}\phi -\sin^{2}\phi ).$$

Combining these equations gives

$$\tau_{xx}=\mu \left(\varepsilon_{xx}\sin^{4}\phi +\varepsilon_{yy}\sin^{2}\phi \cos^{2}\phi -\varepsilon_{xy}2\sin^{3}\phi \cos\phi \right)$$

$$\tau_{yy}=\mu \left(\varepsilon_{xx}\sin^{2}\phi \cos^{2}\phi +\varepsilon_{yy}\cos^{4}\phi -\varepsilon_{xy}2\sin\phi \cos^{3}\phi \right)$$

$$\tau_{xy}=-\mu \left(\varepsilon_{xx}\sin^{3}\phi \cos\phi +\varepsilon_{yy}\sin\phi \cos^{3}\phi -\varepsilon_{xy}2\sin^{2}\phi \cos^{2}\phi \right),$$

which is consistent with the expression derived by Wijesinghe and Roth [39].

As seen in Figure 1b, the fibers rotate in both clockwise and counterclockwise directions, and we must consider a mixture of the two. This will eliminate any terms with factors of sinϕ or $\sin^{3}\phi$, as follows:

$$\tau_{xx}=\mu \left(\varepsilon_{xx}\sin^{4}\phi +\varepsilon_{yy}\sin^{2}\phi \cos^{2}\phi \right)$$

$$\tau_{yy}=\mu \left(\varepsilon_{xx}\sin^{2}\phi \cos^{2}\phi +\varepsilon_{yy}\cos^{4}\phi \right)$$

$$\tau_{xy}=\mu \varepsilon_{xy}2\sin^{2}\phi \cos^{2}\phi \;.$$

In the tissue’s frame of reference, we only consider strains in the *y* direction $\left(\varepsilon_{xx}=\varepsilon_{xy}=0\right)$; so,

$$\tau_{yy}=\mu \varepsilon_{yy}\cos^{4}\phi .$$

Finally, in our manuscript, the angle θ is defined as the angle between the fibers and the articular surface; so, $\theta =\phi -\frac{\pi }{2}$. In that case, $\cos^{4}\phi$ = $\sin^{4}\theta$ and

$$\tau_{yy}=\mu \varepsilon_{yy}\sin^{4}\theta .$$

This expression, plus an isotropic contribution, gives Equation (16).
